# Effect of Defects on Piezoelectric Properties of Sm-Doped K_0.47_Na_0.53_NbO_3_ Ceramics

**DOI:** 10.3390/ma18081760

**Published:** 2025-04-11

**Authors:** Pengkun Wu, Dandan Wang, Fengzi Zhou, Yongpeng Ren, Junhu Zhang, Guozhong Zang, Xiaofei Wang, Xingzhong Cao

**Affiliations:** 1 School of Physics and Engineering, Henan University of Science and Technology, Luoyang 471023, China; 220320090796@stu.haust.edu.cn (P.W.); zfz0310@126.com (F.Z.); zhangjunhuwdwl3@126.com (J.Z.); zangguozhong@sina.com (G.Z.); xfw628@163.com (X.W.); 2 School of Materials Science and Engineering, Henan University of Science and Technology, Luoyang 471023, China; ren_yp123@163.com; 3 Institute of High Energy Physics, CAS, Beijing 100049, China

**Keywords:** KNN-based lead-free ceramics, spark plasma sintering, positron annihilation lifetime spectrum, vacancy-like defect

## Abstract

Rare earth element (Sm)-doped potassium sodium niobate (KNN)-based ceramics are fabricated using spark plasma sintering method and their properties are investigated. The results show that all the samples crystallize in a typical perovskite structure with a single orthorhombic phase. With increasing the Sm doping, the ceramics gradually shift toward the relaxor ferroelectric state and the value of dielectric loss angle tangent (*tanδ*) is smaller than 0.05 for *x* ≤ 0.003 ceramic samples. Meanwhile, the optimized piezoelectric charge coefficient *d*_33_ = 128 pC/N, and piezoelectric voltage coefficient *g*_33_ = 18.9 × 10^−3^ Vm/N are obtained when *x* = 0.001. Compared with the undoped sample, the *d*_33_ of *x* = 0.001 ceramics has been significantly enhanced by 28%. The positron annihilation lifetime results indicate that the main defect types in the ceramics are the A-site vacancies and defect dipoles. Based on the aforementioned results, the optimized piezoelectric performance and the lowest defect dipoles concentration in *x* = 0.001, may be attributed to the low internal oxygen vacancy concentration in it. This work may provide insights for the further study of KNN-based piezoelectric ceramics.

## 1. Introduction

Since the first discovery of piezoelectricity in tourmaline by Pierre Curie, piezoelectric materials, in which an electric field can be induced by the mechanical strain or vice versa, have been extensively studied because of the significant applications including sensor, transducer and actuator [1,2]. Currently, lead-based piezoelectric ceramic materials such as lead zirconate titanate (PZT) have been widely used due to their excellent electrical properties and temperature stability. However, with the increasing demand for healthy living and the growing awareness of environmental protection, lead-free piezoelectric materials are bound to become a trend for piezoelectric materials research [3]. Compared with materials such as BaTiO_3_, SrTiO_3_, and CaTiO_3_, which also have a perovskite structure, KNN-based piezoelectric ceramics possess a relatively high Curie temperature and a moderate piezoelectric constant. Therefore, they are regarded as one of the promising candidate materials to replace PZT ceramics [4,5,6].

At present, the piezoelectric constant *d*_33_ of KNN ceramics sintered by traditional solid-state reaction method is only about 80 pC/N, which still has a significant performance gap compared to lead-based ceramics [7,8]. To meet the even-increasing demands of the advanced piezoelectric devices, enormous efforts have been denoted to improve the electrical properties of KNN-based ceramics, and significant achievements have been made in recent years. For example, in 2004, Satio et al. [9] used the reaction templated grain in growth (RTGG) method to prepare Li, Ta, Sb co-doped KNN-based textured ceramics with orthorhombic-tetragonal phase coexistence. Their ultra-high piezoelectric properties (*d*_33_~416 pC/N) attracted widespread attention. Since then, researchers have begun to improve the piezoelectric properties of KNN-based ceramics by ion doping [10], constructing second components [11], and improving preparation processes [12].

Research has shown that doping with rare earth ions is an effective way to improve the performance of KNN ceramic systems [13,14]. Additionally, the introduction of lanthanide ions can reduce the volatilization of alkali metals such as Na and K in the KNN ceramic system [15], thereby improving the electrical properties of the ceramics. Spark plasma sintering (SPS), as a novel preparation technique with the advantages of rapid heating rate and short soaking time [16], has been more widely used in the fabrication of functional materials including metallic, ceramic, and composite materials. For example, Li et al. [17] achieved a density of 99% of the theoretical density for KNN ceramics prepared using SPS technology.

Positron annihilation lifetime spectrum (PALS) technology works on the principle of detecting the annihilation lifetimes of positrons at various defects within a sample [18], thereby reflecting the types and concentration information of defects. It is a non-destructive testing technique [19,20], and can realize in situ measurement. Consequently, by conducting positron annihilation lifetime studies on the prepared ceramic samples, we expect to correlate the variations in internal vacancy defects with macroscopic electrical properties, which may provide a new approach for regulating the piezoelectric properties of KNN-based ceramics.

Based on the consideration mentioned above, in this paper, lead-free piezoelectric ceramics of (K_0.47_Na_0.53_)_1–3*x*_Sm*_x_*NbO_3_ are prepared by SPS method. The microstructures, electrical properties, and positron annihilation lifetime of the ceramics are characterized, and the influence of internal defects on the piezoelectric properties is systematically studied.

## 2. Materials and Methods

### 2.1. The Preparation of KNSN Ceramics: Spark Plasma Sintering Method

(K_0.47_Na_0.53_)_1–3*x*_Sm*_x_*NbO_3_ ceramic samples were prepared by SPS method. Firstly, the stoichiometric amount of Na_2_CO_3_ (99.99%), K_2_CO_3_ (99.99%), Nb_2_O_5_ (99.9%), Sm_2_O_3_ (99.99%) powders were weighed and milled for 10–12 h in polyethylene jar with different sizes of ZrO_2_ balls, using ethanol as a medium. After being mixed well in a planetary ball mill, the slurry was taken out and dried in an oven and calcined at 850 °C for 6 h to ensure the formation of a single phase niobate. The calcined powder was re-milled for 12 h to obtain the fine powder. Subsequently, the as-obtained powder was loaded into a SPS graphite die (15 mm in diameter) which was fixed and sealed in the chamber. After evacuated under the vacuum of ~10^−2^ Pa, the chamber was firstly heated to 800 °C at a rate of 100 °C/min, then to 900 °C at a rate of 20 °C/min and kept at that temperature for 5 min, with a constant pressure of 30 MPa applied along the Z-axis.

### 2.2. Characterization and Methods

The crystal structure of the ceramic samples was characterized using an X-ray diffraction (XRD, D8 Advance, Bruker, Karlsruhe, Germany) with Cu Kα (λ = 1.5406 Å). The microscopic morphology, elemental distribution, and chemical composition of the samples were measured using the scanning electron microscopy equipped with an energy dispersive spectroscopy unit (SEM-EDS, Crossbeam 350, Carl Zeiss, Oberkochen, Germany). The bulk density of ceramics was measured by Archimedes technique. For the measurements of the electrical properties of ceramics, the electrodes were prepared by the silver paste coated on the upper and lower surfaces of the sample and sintered at 550 °C for 30 min. The temperature dependence of the dielectric properties of ceramic samples was measured using an impedance analyzer (Agilent 4294A, Agilent, Santa Clara, CA, USA) within the temperature range of 30–500 °C. The ferroelectric hysteresis loops were measured at a frequency of 100 Hz at room temperature using the ferroelectric analyzer (TF Analyzer 2000; aixACCT, Microtrac, Aachen, Germany). For the *d*_33_ measurements, the sample was firstly placed in silicone oil and poled under 20 kV/cm bias for 30 min, then measured using a quasi-static piezoelectric constant testing meter (ZJ-3A, Institute of Acoustics, Chinese Academy of Sciences, Beijing, China).

To obtain more-detained defect information in our ceramic samples, we performed the PALS technology on all the ceramic samples. The PALS was performed in the traditional “fast-fast” coincidence method, using a ^22^Na source on a kapton substrate as the positron emission source, and placing the source between two identical samples to form a sandwich-like structure. To ensure the reliability of experimental data, the total of each lifetime spectrum is up to 1.0 × 10^6^. The corresponding lifetime spectra were analyzed by three lifetime fittings using the PATFIT method.

## 3. Results and Discussion

### 3.1. Phase Formation and Microscopic Morphology

**Figure 1:**
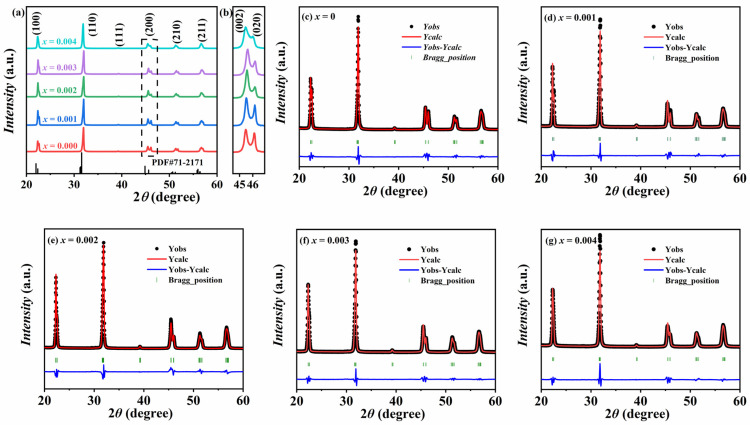
XRD pattern of KNSN ceramics (**a**) 2 = 20°–60°; (**b**) 2*θ* = 44°–47°; (**c**–**g**) Rietveld refinement patterns of all samples.

Figure 1 shows the XRD patterns of the KNSN ceramics measured at room temperature in the range of 20°–60°. From Figure 1a, all samples have typical perovskite structure without the formation of a second phase (PDF#71-2171), indicating that Sm^3+^ has diffused into the KNN lattice and formed a uniform solid solution. Figure 1b shows the local magnification of the (200) diffraction peak of KNSN ceramics near 2*θ* = 45°. The (200) peak splits into two peaks corresponding to (002) and (020), and both peaks display a left-high and right-low pattern. As the doping amount increases, the peak position initially shifts towards a lower angle and subsequently moves to a higher angle. This phenomenon indicates that the unit cell undergoes an initial contraction followed by expansion. Furthermore, the peak intensity ratio of *I*_(002)_ to *I*_(020)_ is approximately 2:1, which preliminarily verifies that all samples possess an orthorhombic phase structure [21,22].

The Rietveld refinement of all samples was carried out using the Fullprof software based on the O-phase (Amm2) model to determine the lattice parameters. The refinement results and relevant parameters are shown in Figure 1c–g and Table 1. The low values of *χ*^2^ and *R_wp_* for all samples indicate that the fitting results are reliable. As can be seen from the table, the phase composition of all fitting results is 100%, which means that all the doped samples are in the O-phase at room temperature. With the increase in the doping amount, the cell volume first decreases and then increases, which is consistent with the variation trend of the (200) diffraction peak. Regarding the broadening of the (200) diffraction peak, the samples with *x* = 0.001–0.003 show a degree of broadening that is basically consistent with that of the undoped sample. The sample with *x* = 0.004 exhibits the most significant peak broadening, which may be related to the presence of a relatively large number of defects inside the ceramic and the refinement of the grains.

**Figure 2:**
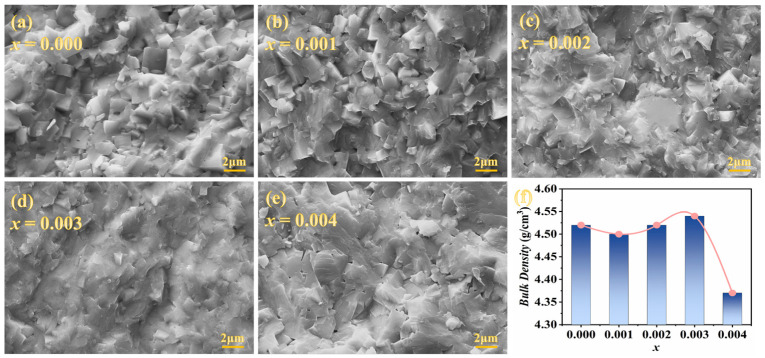
(**a**–**e**) SEM images of KNSN ceramic samples; (**f**) bulk density of KNSN.

Figure 2a–e shows the SEM micrographs of fractured surfaces of the KNSN ceramics. It can be seen that the grains of all samples exhibit a typical cubic shape and are arranged relatively densely. Compared to the sample with *x* = 0.001, some small grains appeared in the ceramics with *x* ≥ 0.002, which filled the voids between large grains and improved the density of the ceramics. This may be due to the donor ion doping feature of Sm^3+^ on the A-site. When Sm^3+^ replaces the A-site ions (K^+^ or Na^+^), cationic vacancies occur to maintain the charge balance within the ceramics. Vacancy-type defects concentrated near the grain boundaries usually exert a “pinning effect” on the movement of grains and inhibit the growth of grains [6,23]. For the sample with *x* = 0.004, some grains fused together, probably due to the more introduction of Sm^3+^, which caused the theoretical sintering temperature of the KNSN ceramics to be lower than the actual sintering temperature, resulting in the formation of liquid phase inside the ceramics. Excessive liquid phase accumulation at grain boundaries can inhibit grain growth and lead to the appearance of more voids, which may be the reason for the sharp decrease in density of the *x* = 0.004 sample [24].

**Figure 3:**
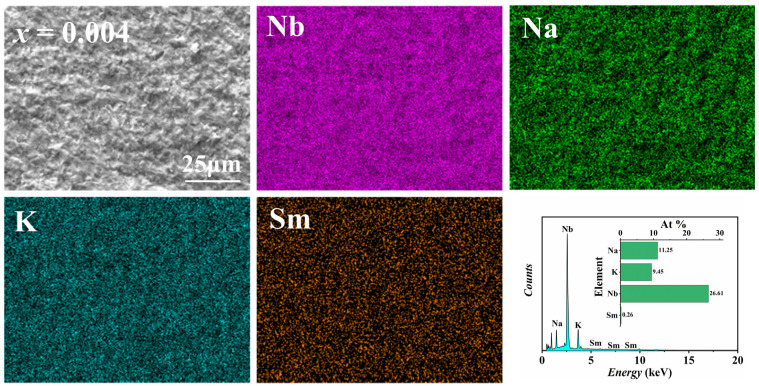
The SEM images and EDS elemental mappings of 0.004 Sm.

In order to analyze the variation in elemental species and contents within each sample at different doping amounts, EDS was used to test the composition and contents of all KNSN ceramic samples. Figure 3 shows the SEM-EDS plot for *x* = 0.004 doping. From the figure, it can be seen that there is no agglomeration or segregation of the four main elements in the observed area, indicating that each element is evenly distributed within the matrix.

Table 2 shows the major element content (at.%) of each sample. For the undoped KNN ceramics, the K/Na ratio deviates from the theoretical value, which may be attributed to the volatilization of alkali metals during sintering as well as annealing. At the same time, the addition of Sm^3+^ increases the K/Na ratio, indicating that Sm^3+^ tends to preferentially replace Na^+^ at the A-site. This can be explained by the ionic radius: the ionic radius of Sm^3+^ (1.24 Å, CN = 12) is close to Na^+^ (1.39 Å, CN = 12) in the A-site, but significantly different from K^+^ (1.64 Å, CN = 12). Therefore, most Sm^3+^ tend to replace Na^+^ first, and then K^+^-site [25].

### 3.2. Dielectric and Relaxation Properties

**Figure 4:**
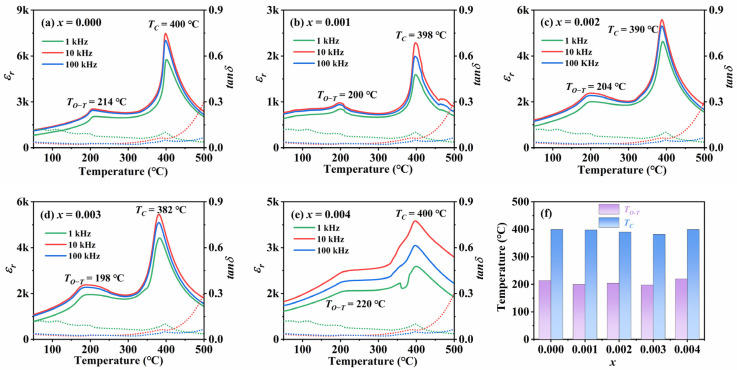
(**a**–**e**) Temperature-dependence of dielectric constant and the value of dielectric loss angle tangent of KNSN ceramics; (**f**) *T_O−T_* and *T_C_* as a function of *x*.

Figure 4a–e shows the variation curves of the dielectric constant and the value of dielectric loss angle tangent of KNSN ceramics with temperature at different frequencies in the range of 1 kHz–100 kHz. It can be clearly seen from the figures that two abrupt peaks appear in the dielectric–temperature curves of all ceramic samples. This indicates that with the increase in temperature, two phase transitions occur inside the KNSN ceramics: the orthorhombic phase (O) to the tetragonal phase (T) and the tetragonal phase (T) to the cubic phase (C). The phase transition temperature of the orthorhombic-tetragonal phase (*T_O−T_*) of all ceramic samples is about 210 °C, and the Curie temperature (*T_C_*) is about 400 °C, indicating that all samples are in the orthorhombic phase near room temperature, which confirms the speculation made in the previous XRD analysis. From Figure 4f, compared with the undoped KNN sample (*T_O−T_*~214 °C, *T_C_*~400 °C), the *T_O−T_* and *T_C_* of the doped samples exhibit an overall decreasing trend, but the temperature variation is within 20 °C, indicating that the introduction of Sm^3+^ has a small impact on the *T_O−T_* and *T_C_*.

Table 3 shows the dielectric parameters of each sample at 1 kHz in room temperature. Among all the samples, *x* = 0.004 samples have the highest relative dielectric constant of 892, which is about 17% higher than the undoped ceramics. In addition, the value of dielectric loss angle tangent of *x* ≤ 0.003 samples is kept at a low level (<0.05). On the one hand, the improvement in the dielectric properties can be attributed to the introduction of the more rare earth element Sm^3+^, which replaces A-site ions and creates more cationic vacancies in the ceramics. The existence of these vacancies makes the domain walls easier to move under the action of an electric field or external force. The increase in the mobility of domain walls means that the polarization of the material is more likely to change under the action of an electric field, thus leading to an increase in the dielectric constant [26,27]. On the other hand, low dielectric loss may be due to the advantages of the SPS preparation process: compared with the traditional sintering process, the SPS process can lower the volatilization amount of Na and K elements by approximately 30–50%. Meanwhile, for the ceramic samples sintered by the SPS process, their density can usually reach 95–99% of the theoretical density [16].

**Figure 5:**
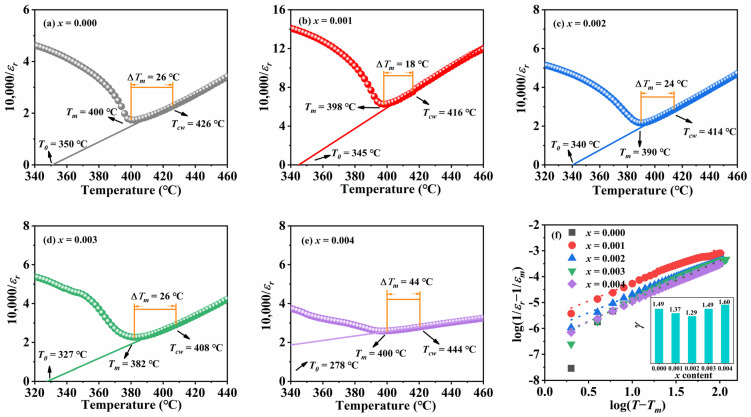
(**a**–**e**) The reciprocal of the dielectric constant of KNSN ceramics and temperature; (**f**) log(1εr−1εm) vs. log(T−Tm) (illustrated as the variation in diffusion factor with *x*).

As shown in Figure 4a–e, the T-C phase transition temperature range of the ceramics gradually expands with the increase in the doping amount, indicating that the relaxation characteristics of the ceramic samples have changed. The reason for the expansion of the temperature range of T-C phase transition may be that, after doping ions enter the ceramic matrix, local electric fields are generated around them due to the difference in charge and size between them and the matrix ions. The local electric field affects the behavior of electric domains, allowing phase transitions to occur over a wider temperature range, thereby expanding the range of T-C phase transitions. Meanwhile, if there is no significant structural damage or sudden change in performance during the phase transition process, the material can maintain a relatively stable structure and performance at different temperatures, and its thermal stability is improved. For example, for samples with *x* ≤ 0.03 in this article, the T-C phase transition temperature range widens, but the internal crystal structure of the ceramic does not undergo significant changes. Therefore, appropriate doping of Sm may improve the application of KNN ceramics in different temperature environments [26]. Figure 5a–e shows the relationship between the reciprocal of relative dielectric constant and temperature at 1 kHz, which is used to investigate the effect of Sm^3+^ introduction on the relaxation characteristics of KNN-based ceramics.

For normal ferroelectrics, the dielectric constant follows the Curie–Weiss law:

(1) $$\begin{array}{c} \frac{1}{\varepsilon }=\frac{T-T_{cw}}{C}\;\left(T>{\;T}_{c}\right) \end{array}$$

The degree of deviation of ceramics from the Curie–Weiss law, Δ*T*, is expressed as

(2) $$\begin{array}{c} \Delta T_{m}=T_{cw}-T_{m} \end{array}$$

where *C* is the Curie–Weiss constant and *T_cw_* is the Curie–Weiss temperature. *T_cw_* is the temperature corresponding to the point at which *ε_r_* starts to follow the Curie–Weiss law, and *T_m_* is the temperature corresponding to the maximum value of *ε_r_*. From the figure, it can be seen that Δ*T* continues to increase in Sm-doped ceramics, indicating that the ceramic samples increasingly deviate from the Curie–Weiss law and the diffusion phase transition behavior is gradually enhanced.

The relative dielectric constant above *T_m_* is described by the modified Curie–Weiss law:

(3) $$\begin{array}{c} \frac{1}{\varepsilon }-\frac{1}{\varepsilon_{m}}=\frac{{\left(T-T_{c}\right)}^{\gamma }}{C} \end{array}$$

where *ε_m_* is the maximum value of the relative constant at the transition temperature *T_m_*, *T_C_* is the Curie temperature and *γ* is the diffusion coefficient. The log (1*/ε_r_* − 1*/ε_m_*) vs. log (*T* − *T_m_*) curve is plotted according to the modified Curie–Weiss law, and the slope obtained from a linear fit to the curve is the diffusion factor *γ*. For a normal ferroelectric material, *γ* = 1, and for a standard relaxor ferroelectric material, *γ* = 2 [28]. 

As shown in Figure 5f, the *γ* value gradually increases from 1.37 to 1.6 with the increase in the doping amount, which further indicates that the relaxation behavior of KNSN ceramics is enhanced by the Sm doping. The larger the value of *γ*, the more obvious the relaxor characteristics of the material are. This is because there are various microscopic inhomogeneities inside the material, such as fluctuations in chemical composition, cation vacancies, etc., which lead to the enhancement of polarization fluctuations. The size of the ferroelectric domains becomes smaller and their distribution becomes more disordered, thus making the polarization behavior exhibit relaxor characteristics [29].

### 3.3. Ferroelectric and Piezoelectric Properties

**Figure 6:**
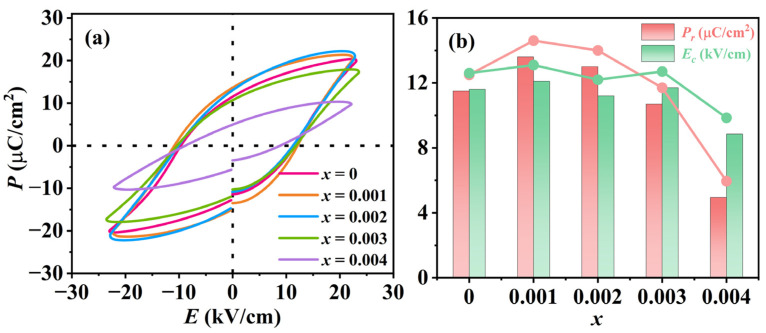
(**a**) Polarization–electric field (*P*–*E*) hysteresis loops; (**b**) the *P_r_* and *E_c_* of KNSN ceramics.

Figure 6a shows the polarization–electric field (*P*–*E*) hysteresis loop of KNSN ceramic samples at *E* = 20 kV/cm, and all ceramic samples exhibit typical ferroelectric characteristics. The hysteresis loop of the sample with *x* = 0.004 is unsaturated, which may be attributed to the presence of numerous internal voids and poor densification in the ceramics, resulting in insufficient flipping of the electric domains during the polarization process [30]. Figure 6b depicts the relationship between remnant polarization *P_r_*, coercive field *E_C_*, and doping amount *x*. As the doping amount increases, *P_r_* exhibits a tendency to increase and then decrease, and achieves the maximum value at *x* = 0.001 (*P_r_* = 13.6 μC/cm^2^).

**Figure 7:**
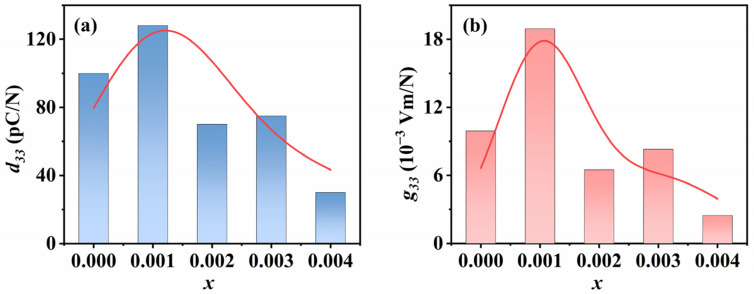
The variation in (**a**) piezoelectric strain constant *d*_33_; (**b**) piezoelectric voltage constant *g*_33_.

Figure 7 shows the piezoelectric properties of KNSN ceramic samples. The *d*_33_ and *g*_33_ are both important parameters in characterizing piezoelectric ceramics for practical applications. And *d*_33_ can be directly measured, while *g*_33_ is calculated using the following formula:

(4) $$\begin{array}{c} g_{33}=\frac{d_{33}}{\varepsilon_{r}\varepsilon_{0}} \end{array}$$

where *ε_r_* is the relative dielectric constant and *ε*_0_ is the vacuum dielectric constant. As can be seen from Figure 7, the values of *d*_33_ and *g*_33_ for all ceramic samples exhibit a similar trend, reaching their maximum values at *x* = 0.001 (*d*_33_ = 128 pC/N, *g*_33_ = 18.9 × 10^−3^ Vm/N). This result also indicates that the ceramics with *x* = 0.001 doping level have more outstanding overall piezoelectric properties.

Based on the above analysis, we speculate that the factors influencing the electrical properties of ceramics include the following aspects. The sample with *x* = 0.001 shows the most excellent piezoelectric properties, which may be attributed to its larger remnant polarization and grain size, facilitating domain switching and domain wall motion. However, for samples with *x* ≥ 0.002, the piezoelectric properties of the ceramics show different degrees of degradation. It may be attributed to the fact that more Sm^3+^ doping leads to the emergence of many small grains in the KNN ceramics, and the small grains will increase the grain boundaries, enhance the coupling effect between grains, and impede the movement of the domain walls, which leads to the degradation of the piezoelectric properties of the samples [31,32]. 

### 3.4. Positron Annihilation Lifetime Spectroscopy

In general, A-site donor doping can improve the properties of KNN-based ceramics such as piezoelectricity and dielectricity [6], but the results of this study do not fully align with this viewpoint. Therefore, we would like to explain the corresponding results by investigating the defect changes inside the ceramics. PALS has a unique advantage in detecting internal defects as well as concentration distributions in materials due to the ability of positrons to sensitively reflect the electron density information at different locations [33]. Therefore, we use PALS technology to characterize the defect characteristics inside ceramic samples. 

Here, we perform the three-components fit on all ceramic samples. Since the intensity of the longest lifetime component *τ*_3_ (2–3 ns) is less than 3%, it is considered to be the result of positron annihilation on the source and sample surfaces and is therefore not taken into account in the analysis. Table 4 shows the results of the normalized positron annihilation lifetimes, where *I*_1_ and *I*_2_ are the intensities corresponding to the first lifetime component *τ*_1_ and the second lifetime component *τ*_2_, respectively. From the table, it can be seen that the *τ*_1_ values are basically around 157–160 ps. According to relevant theoretical calculations, the annihilation lifetime of positrons in a perfect KNN lattice is 157 ps [34]. Therefore, we assume that *τ*_1_ corresponds to the free-state annihilation lifetime of positrons in KNSN ceramics.

**Figure 8:**
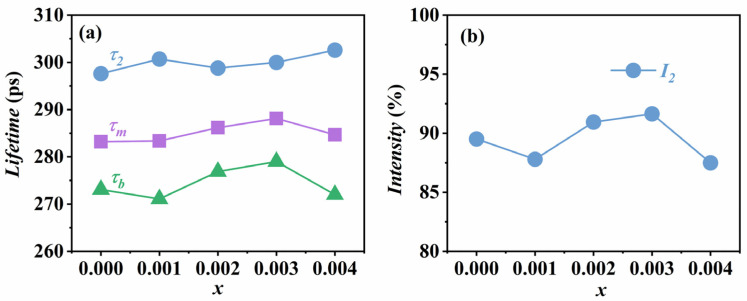
(**a**) Variation of *τ*_2_, *τ_m_*, and *τ_b_* with doping amount; (**b**) intensity *I*_2_ corresponding to *τ*_2_.

Figure 8a shows the variation in the second lifetime *τ*_2_, the average lifetime *τ_m_*, and the bulk lifetime *τ_b_* with the doping amount obtained from the three-components fitting. The values of *τ_m_* and *τ_b_* are calculated using the following formula:

(5) $$\begin{array}{c} \tau_{m}=\tau_{1}I_{1}\times {\;\tau }_{2}I_{2} \end{array}$$

(6) $$\begin{array}{c} \tau_{b}=\frac{\tau_{1}\times \tau_{2}}{\tau_{1}I_{2}+\tau_{2}I_{1}} \end{array}$$

From Figure 8a, we can see that the average lifetime of all samples is higher than the bulk lifetime, indicating that there are vacancy defects in the ceramics that can trap positrons [35]. Moreover, the value of *τ_m_* fluctuates around 285 ps with a small range of variation, so it can be assumed that the information about defects in the interfacial region inside the ceramics has not been changed. Since *τ*_1_ is considered as the free-state annihilation lifetime of positrons in ceramics, then *τ*_2_ reflects the type of defects inside the ceramics. According to theoretical calculations, the annihilation lifetime of positrons in the A-site cation vacancy *V_A_* is around 253 ps, and the presence of the defect dipole *V_A-O_* formed by the A-site vacancy and the oxygen vacancy will increase this value [34]. For example, the theoretical value of the annihilation lifetime of positrons in *V_A_* + 4*V_O_* is about 280 ps, while the value of *τ*_2_ obtained in our experiments is around 300 ps, so we consider that some *V_A-O_* exist in the prepared ceramic samples in addition to *V_A_*.

The variation of *V_A-O_* concentration is determined by analyzing the intensity *I*_2_ corresponding to *τ*_2_, and the trends and values are shown in Figure 8b and Table 4, respectively. For the undoped KNN sample, its *I*_2_ is about 89.5%, indicating that some *V_A_* and *V_A-O_* are already present within the sample. This is due to the volatilization of alkali metals and the loss of oxygen elements during the sintering and annealing processes of the sample [23], which can be expressed as

(7) $$\begin{array}{c} Na_{Na}+e^{\prime }\;\to \;Na+{\;V}_{Na}^{\prime } \end{array}$$

(8) $$\begin{array}{c} K_{K}+e^{\prime }\;\to \;K+V_{k}^{\prime } \end{array}$$

(9) $$\begin{array}{c} O_{O}\;\to \;\frac{1}{2}\;\left(O_{2}\right)+V_{0}^{\cdot \cdot }+2e^{\prime } \end{array}$$

Comparing with the *x* = 0.002 and *x* = 0.003 samples, the *I*_2_ of the *x* = 0.001 sample is lower, about 87.8%, which illustrates that the concentration of *V_A-O_* in the sample is lower. This may be due to the lower concentration of oxygen vacancies inside the ceramic, which reduces the probability of forming defect dipoles. For the sample with *x* = 0.004, the *I*_2_ is about 87.5%, probably because of more A-site vacancies inside the ceramic due to the increase in Sm doping. Since the radius of Sm^3+^ is much larger than the radius of the B-site (Nb^5+^) ions, the probability of replacing the B-site ions is low, and therefore the concentration of B-site vacancies will not change significantly. According to the Schottky defect equation, $nil{\Leftrightarrow \;V}_{A}^{\prime \prime }+3V_{B}^{\prime \prime \prime \prime }+V_{o}^{\cdot \cdot }$, when the A-site vacancies increase, in order to maintain the defect equilibrium constant $\kappa_{V_{o}^{\prime \prime }}=\left[V_{A}^{\prime \prime }\right]\left[V_{B}^{\prime \prime \prime \prime }\right]{\left[V_{o}^{\cdot \cdot }\right]}^{3}$ unchanged, the concentration of oxygen vacancies will inevitably decrease [36], resulting in a lower concentration of *V_A-O_* formed.

The PALS results reveal that the changes in the electrical properties of KNN ceramics caused by Sm^3+^ doping are attributed to the changes in A-site vacancies and oxygen vacancies. For example, the *x* = 0.001 sample exhibits the best piezoelectric performance, which may be ascribed to the high number of A-site cation vacancies and the low concentration of oxygen vacancies in the sample, thus reducing the probability of forming defect dipoles. This makes domain switching and domain wall motion easier, resulting in better piezoelectric performance; when *x* ≥ 0.002, with the increase of oxygen vacancies inside the ceramic, the probability of forming defect dipoles between A-site vacancies and oxygen vacancies increases [37], which enhances the pinning effect on domain inversion and domain wall motion. At the same time, the appearance of small grains increases the coupling effect between grains, ultimately leading to the deterioration of ceramic piezoelectric properties.

## 4. Conclusions

In this work, (K_0.47_Na_0.53_)_1–3*x*_Sm*_x_*NbO_3_ ceramics are successfully prepared by the spark plasma sintering method, the electrical properties and the positron annihilation lifetime spectroscopy of the ceramics are investigated. The best piezoelectric performance of the ceramics is obtained at the *x* = 0.001 doping, with a piezoelectric constant of 128 pC/N, which is 28 % higher than that of the undoped ceramics. The introduction of Sm improves the density of ceramics to a certain extent, but also leads to the creation of small grains, and the coupling effect between the small grains leads to the degradation of the electrical properties of ceramics. The PALS results reveal that the change in the electrical properties of the KNSN ceramic system may be related to the presence of cationic vacancies and defect dipoles within it. The defect dipoles at low concentrations exhibit a weaker pinning effect on domain wall motion and domain switching, thereby enhancing the piezoelectric properties of ceramics. Therefore, this paper has investigated the relation between internal defects and macroscopic properties of KNN-based ceramics by PALS technique, which may provide some new views to study the origin of high piezoelectric performance of KNN-based ceramics.

## Figures and Tables

**Table 1:** Rietveld refinement parameters of KNSN piezoelectric ceramics.

x	0 Sm	0.001 Sm	0.002 Sm	0.003 Sm	0.004 Sm
O-Phase (%)	100	100	100	100	100
*a* (Å)	3.9477	3.9478	3.9469	3.9473	3.9543
*b* (Å)	5.6354	5.6332	5.6289	5.6258	5.6336
*c* (Å)	5.6616	5.6624	5.6543	5.6526	5.6538
*α* = *γ* = *β*	90°	90°	90°	90°	90°
Volume (Å^3^)	125.956	125.926	125.692	125.526	125.949
*χ* ^2^	4.33	5.96	3.85	4.17	6.25
*R_wp_*	8.52	9.13	9.66	10.1	8.78

**Table 2:** Element content in KNSN ceramic samples.

Element (at%)	K	Na	O	Nb	Sm	K/Na Atomic Ratio
0 Sm	8.40	10.84	61.87	18.92	0	0.77
0.001 Sm	9.87	10.72	52.46	26.84	0.13	0.92
0.002 Sm	7.61	8.42	51.19	21.67	0.14	0.90
0.003 Sm	10.07	10.69	54.86	24.20	0.17	0.94
0.004 Sm	9.45	11.25	52.43	26.61	0.26	0.84

**Table 3:** KNSN ceramic dielectric properties parameters at 1 kHz in room temperature.

Samples	*ε_r_*	*tanδ*	*T_O−T_*	*T_C_*
0 Sm	762	0.037	214 °C	400 °C
0.001 Sm	604	0.032	200 °C	398 °C
0.002 Sm	887	0.038	204 °C	390 °C
0.003 Sm	717	0.041	198 °C	382 °C
0.004 Sm	892	0.092	220 °C	400 °C

**Table 4:** Results of normalized PALS solution spectra of KNSN ceramics.

Samples	*τ*_1_ (ps)	*I*_1_ (%)	*τ*_2_ (ps)	*I*_2_ (%)	*τ_m_* (ps)	*τ_b_* (ps)
0 Sm	160.3	10.49	297.6	89.51	283.2	273.1
0.001 Sm	158.7	12.20	300.7	87.80	283.4	271.1
0.002 Sm	159.4	9.05	298.8	90.95	286.2	276.9
0.003 Sm	157.9	8.36	300.0	91.64	288.1	279.0
0.004 Sm	159.3	12.51	302.6	87.49	284.7	272.0

## Data Availability

The original contributions presented in this study are included in the article. Further inquiries can be directed to the corresponding authors.

## References

[1] Liu T., Zheng Z., Li Y., Jia P., Wang Y. (2022). Improved comprehensive properties induced by multi-phase coexistence in KNN ceramics. Mater. Chem. Phys..

[2] Wang Z., Ma D., Wang Y., Xie Y., Yu Z., Cheng J., Li L., Sun L., Dong S., Wang H. (2023). Kirigami-origami-inspired lead-free piezoelectric ceramics. Adv. Sci..

[3] Lee G.-S., Kim J.-S., Kim S.-H., Kwak S., Kim B., Kim I.-S., Nahm S. (2024). Recent developments in (K,Na)NbO_3_-based lead-free piezoceramics. Micromachines.

[4] Pan Q., Pu Y., Wang B., Xie H., Zhang L., Zhang J., Hao Y., Yang Y., Qian J. (2025). Substantial increase in resistance and suppression of resistance degradation in SrTiO_3_-based ceramics with colossal permittivity and low dielectric loss. Ceram. Int..

[5] Ali S.M., Shankar J., Kumar A.S., Raju P. (2023). Effect of sintering temperature on physical and dielectric properties of SrTiO_3_ ceramics. Mater. Today Proc..

[6] Clabel J.L., Paula K.T., Pereira-da-Silva M.A., Vollet-Filho J.D., Marega E., Mendonça C.R. (2023). Fabrication of micro patterns on BaTiO_3_:Er^3+^/Yb^3+^ perovskite films by femtosecond laser micromachining. Appl. Surf. Sci..

[7] Lv X., Zhu J., Xiao D., Zhang X., Wu J. (2020). Emerging new phase boundary in potassium sodium-niobate based ceramics. Chem. Soc. Rev..

[8] Trolier-McKinstry S., Zhang S., Bell A.J., Tan X. (2018). High-performance piezoelectric crystals, ceramics, and films. Annu. Rev. Mater. Res..

[9] Saito Y., Takao H., Tani T., Nonoyama T., Takatori K., Homma T., Nagaya T., Nakamura M. (2004). Lead-free piezoceramics. Nature.

[10] Miwa Y., Hayashi H., Kawada S., Tanaka N. (2023). The dielectric and piezoelectric properties of CaZrO_3_-modified (K,Na)NbO_3_ bulk and multilayer ceramics with different ZrO_2_ additions and MnO content. Jpn. J. Appl. Phys..

[11] Li H., Hao Y., Lin Z., He X., Cai J., Gong X., Gu Y., Zhang R., Cheng H., Zhang B. (2022). (K,Na)NbO_3_ lead-free piezoceramics prepared by microwave sintering and solvothermal powder synthesis. Solid State Commun..

[12] Wu Y., Cheng Y., Guan S., Wang X., Shi W., Xu H., Lang R., Xing J., Zhu J., Chen Q. (2023). KNN-based lead-free piezoelectric ceramics with high Q_m_ and enhanced d_33_ via a donor-acceptor codoping strategy. Inorg. Chem..

[13] Vendrell X., García J., Cerdeiras E., Ochoa D., Rubio-Marcos F., Fernández J., Mestres L. (2016). Effect of lanthanide doping on structural, microstructural and functional properties of K_0.5_Na_0.5_NbO_3_ lead-free piezoceramics. Ceram. Int..

[14] Zhang Z., Shang X., Liu X., He Y., Zhang Z., Guo J. (2024). Achieving excellent mechanical and electrical properties in transition metal oxides and rare earth oxide-doped KNN-based piezoceramics. J. Am. Ceram. Soc..

[15] Hreščak J., Dražić G., Deluca M., Arčon I., Kodre A., Dapiaggi M., Rojac T., Malič B., Bencan A. (2017). Donor doping of K_0.5_Na_0.5_NbO_3_ ceramics with strontium and its implications to grain size, phase composition and crystal structure. J. Eur. Ceram. Soc..

[16] Morshed T., Haq E.U., Silien C., Tofail S.A.M., Zubair M.A., Islam M.F. (2020). Piezo and pyroelectricity in spark plasma sintered potassium sodium niobate (KNN) ceramics. IEEE Trans. Dielectr. Electr. Insul..

[17] Li J., Wang K., Zhang B., Zhang L. (2006). Ferroelectric and piezoelectric properties of fine-grained Na_0.5_K_0.5_NbO_3_ lead-free piezoelectric ceramics prepared by spark plasma sintering. J. Am. Ceram. Soc..

[18] Tuomisto F., Makkonen I. (2013). Defect identification in semiconductors with positron annihilation: Experiment and theory. Rev. Mod. Phys..

[19] Zhang L., Wang T., Wang L., Liu J., Zhao M., Ye B. (2012). Structural defects and non-ferroelectric piezoelectricity in an unpoled SrTiO_3_-Bi_12_TiO_20_ (ST-BT) composite ceramics. Scr. Mater..

[20] Nagai Y., Tang Z., Hasegawa M. (2000). Chemical analysis of precipitates in metallic alloys using coincidence Doppler broadening of positron annihilation radiation. Radiat. Phys. Chem..

[21] Zhang Q., Xu F., Yang R., Lu Y., Li P., Shang X., Zhou T., He Y. (2017). Suppressed tanδ and enhanced Q_m_ in KCT and Ni_2_O_3_ co-modified [(K_0.43_Na_0.57_)_0.94_Li_0.06_] [(Nb_0.94_Sb_0.06_)_0.95_Ta_0.05_O_3_ lead-free piezoelectric ceramics. Ceram. Int..

[22] Zhang Y., Shen B., Zhai J., Zeng H. (2016). New insight on sintering progress of KNN-based lead-free ceramics. J. Am. Ceram. Soc..

[23] Shi W., Feng Y., Lu T., Lu Y., Shen J., Xue J., Du J., Fu P., Hao J., Li W. (2019). Photoluminescence and impedance properties of rare-earth doped (K_0.5_Na_0.5_)NbO_3_ lead-free ceramics. J. Mater. Sci. Mater. Electron..

[24] Xiong Y., Wu M., Yang W., Chen W., Wang L., Zhou Z. (2017). Structure, piezoelectric performance and liquid phase aid sintering of SiO_2_ doped (K_0.5_Na_0.5_)NbO_3_ lead-free ceramics. J. Mater. Sci. Mater. Electron..

[25] Yu F., Chi Y., Wang P., Ma B., Wu X., Lin C., Zhao C., Gao M., Lin T., Zhang Q. (2022). Highly responsive photochromic behavior with large coloration contrast in Ba/Sm co-doped (K_0.5_Na_0.5_)NbO_3_ transparent ceramics. Ceram. Int..

[26] Xing J., Xie S., Wu B., Tan Z., Jiang L., Xie L., Cheng Y., Wu J., Xiao D., Zhu J. (2020). Influence of different lanthanide ions on the structure and properties of potassium sodium niobate based ceramics. Scr. Mater..

[27] Liu H., Veber P., Rödel J., Rytz D., Fabritchnyi P.B., Afanasov M.I., Patterson E.A., Frömling T., Maglione M., Koruza J. (2018). High-performance piezoelectric (K,Na,Li)(Nb,Ta,Sb)O_3_ single crystals by oxygen annealing. Acta Mater..

[28] Yang D., Ma C., Yang Z., Wei L., Chao X., Yang Z., Yang J. (2016). Optical and electrical properties of pressureless sintered transparent (K_0.37_Na_0.63_)NbO_3_-based ceramics. Ceram. Int..

[29] Zang J., Li M., Sinclair D.C., Jo W., Rödel J. (2014). Impedance spectroscopy of (Bi_1/2_Na_1/2_)TiO_3_-BaTiO_3_ ceramics modified with (K_0.5_Na_0.5_)NbO_3_. J. Am. Ceram. Soc..

[30] Jiang M., Li X., Liu J., Zhu J., Zhu X., Li L., Chen Q., Zhu J., Xiao D. (2009). Structural and electrical properties of Cu-doped (K_0.5_Na_0.5_)NbO_3_-MgTiO_3_ lead-free ceramics. J. Alloys Compd..

[31] Liu W., Wang H., Hu W., Du Y., Cheng C. (2022). Understanding the origin of the high piezoelectric performance of KNN-based ceramics from the perspective of lattice distortion. Ceram. Int..

[32] Rubio-Marcos F., Fernandez J.F., Ochoa D.A., García J.E., Rojas-Hernandez R.E., Castro M., Ramajo L. (2017). Understanding the piezoelectric properties in potassium-sodium niobate-based lead-free piezoceramics: Interrelationship between intrinsic and extrinsic factors. J. Eur. Ceram. Soc..

[33] Klym H., Karbovnyk I., Luchechko A., Kostiv Y., Popov A.I. (2022). Extended positron-trapping defects in the Eu^3+^-doped BaGa_2_O_4_ ceramics studied by positron annihilation lifetime method. Phys. Status Solidi B.

[34] Espinosa A.P., Ramajo L., Rubio-Marcos F., Macchi C., Somoza A., Castro M. (2021). Influence of the BaTiO_3_ addition to K_0.5_Na_0.5_NbO_3_ lead-free ceramics on the vacancy-like defect structure and dielectric properties. J. Eur. Ceram. Soc..

[35] Wang T., Zhu T., Wang D., Zhang P., Song Y., Ye F., Wang Q., Jin S., Yu R., Liu F. (2022). Effect of vacancy behavior on precipitate formation in a reduced-activation V-Cr-Mn medium-entropy alloy. Materials.

[36] Yang J., Feng S.-R., Zhang T., Niu X.-P., Wang R., Li M., Yu R.-S., Cao X.-Z., Wang B.-Y. (2024). Analysis of defects in B-vacancy compensated Sm-doped PZT(54/46) ceramics and their influences on piezoelectric properties. Acta Phys. Sin..

[37] Sheng Y., Huang Y., Chen C., Zhang M., Deng N., Ma L. (2018). Effect of oriented defect-dipoles on the ferroelectric and piezoelectric properties of CuO-doped (K_0.48_Na_0.52_)_0.96_Li_0.04_Nb_0.805_Ta_0.075_Sb_0.12_O_3_ ceramics. Ceram. Int..

